# Alpha oscillatory correlates of motor inhibition in the aged brain

**DOI:** 10.3389/fnagi.2015.00193

**Published:** 2015-10-13

**Authors:** Marlene Bönstrup, Julian Hagemann, Christian Gerloff, Paul Sauseng, Friedhelm C. Hummel

**Affiliations:** ^1^Brain Imaging and Neurostimulation Laboratory, Department of Neurology, University Medical Centre Hamburg-EppendorfHamburg, Germany; ^2^Department of Psychology, Ludwig-Maximilians-University MunichMunich, Germany

**Keywords:** aging, electroencephalography, inhibition, motor control, alpha rhythm, neuroplasticity

## Abstract

Exerting inhibitory control is a cognitive ability mediated by functions known to decline with age. The goal of this study is to add to the mechanistic understanding of cortical inhibition during motor control in aged brains. Based on behavioral findings of impaired inhibitory control with age we hypothesized that elderly will show a reduced or a lack of EEG alpha-power increase during tasks that require motor inhibition. Since inhibitory control over movements has been shown to rely on prior motor memory formation, we investigated cortical inhibitory processes at two points in time—early after learning and after an overnight consolidation phase and hypothesized an overnight increase of inhibitory capacities. Young and elderly participants acquired a complex finger movement sequence and in each experimental session brain activity during execution and inhibition of the sequence was recorded with multi-channel EEG. We assessed cortical processes of sustained inhibition by means of task-induced changes of alpha oscillatory power. During inhibition of the learned movement, young participants showed a significant alpha power increase at the sensorimotor cortices whereas elderly did not. Interestingly, for both groups, the overnight consolidation phase improved up-regulation of alpha power during sustained inhibition. This points to deficits in the generation and enhancement of local inhibitory mechanisms at the sensorimotor cortices in aged brains. However, the alpha power increase in both groups implies neuroplastic changes that strengthen the network of alpha power generation over time in young as well as elderly brains.

## Introduction

The integration of individuals into modern societies relies on their ability to acquire and control skills in the context of a particular situation. Current trends in the demographics of developed countries show that not only society in general but also the labor force is growing older (Tossi, 2005, 2012). Given this age profile of most western societies and the likely prospect of an increasingly older workforce, it is necessary to better understand changes in cognitive control across the lifespan in order to identify pathways for interventions designed to optimize a person's ability to function in the workplace, and to maximize societal participation and quality of life in older age.

Context-dependent adaptation of learned actions is essential for a person's ability of functioning adequately in the environment characterizing our modern world. This dynamic regulation of stimulus-action associations, also known as *executive behavioral control*, relies heavily on the appropriate acquisition and context-dependent suppression of information and actions (Aron, 2007). Moreover, this ability is required to behave successfully in complex environments such as at work, in social interactions, or during the use of new communication devices and media. Exerting inhibitory control is a cognitive ability mediated by functions known to decline with age (Bedard et al., 2002; Gazzaley and D'Esposito, 2007; Anguera and Gazzaley, 2012).

In motor behavior, inhibitory control of a movement which is learned to be typically executed in a specific context, like picking up the phone when it rings, need to be withheld if the context changes. For example, if one is about to accept the call but the name of the caller appears to be the non-liked mother-in-law, to behave successfully would be that one continuously inhibits the prepared response of accepting the call and lifting the phone to the ear although the phone keeps on ringing. In this situation a learned motor memory trace is inhibited as required by the context of the situation but without time constraints. The cortical correlates of such a sustained inhibitory response are subject to this study.

The physiological basis of behaviorally relevant executive control relies on cortical inhibitory processes resulting in well-orchestrated activation and deactivation of brain regions. The neurophysiological mechanisms of cortical inhibition are of great interest because the pathophysiology of many diseases has been associated with deficient inhibitory capabilities such as essential tremor (Paris-Robidas et al., 2012), dystonia (Hummel et al., 2002; Sohn and Hallett, 2004; Quartarone and Hallett, 2013), attention deficit/hyperactivity disorder (Buchmann et al., 2007; Clark et al., 2007), and seizures (Baroncelli et al., 2011; Kaila et al., 2013). In addition, during healthy aging, altered sensorimotor processing, attention and memory has been related to declining inhibitory capacities (Luria and Ryan, 1973; Hasher et al., 1991; Kramer et al., 1994; McDowd and Filion, 1995; Butler and Zacks, 2006; Glisky, 2007; Bailey and Henry, 2008). Accordingly, neurophysiological parameters indicative of cortical inhibitory processes exhibit gradual age-related alterations (Chao and Knight, 1997; Kok, 1999; Peinemann et al., 2001; Gleichmann et al., 2011; Marneweck et al., 2011; Heise et al., 2013; Bañuelos et al., 2014).

The goal of the present study is to add to the mechanistic understanding of age-related differences in inhibition at the cortical and behavioral level. Specifically in the motor domain, this has not yet been addressed in detail (Grachev et al., 2001; Stagg et al., 2011).

Previous studies have shown that desynchronization in the alpha frequency range represents a correlate of increased cellular excitability in thalamocortical systems (Jasper and Droogleever-Fortuyn, 1948; Steriade and Llinás, 1988). An increase in alpha power was originally related to an idling of cortical areas. Recent observations have challenged this view and have demonstrated its role in inhibitory control processes (Hummel et al., 2002; Lange et al., 2013; Sauseng et al., 2013). Especially in situations where subjects have to withhold or control a response or suppress irrelevant information an increase in alpha power has been observed (Jensen and Mazaheri, 2010). Based on these findings, the framework for an alpha theory of inhibition was formulated (Klimesch et al., 2007). In the motor domain this concept is supported by previous studies with a paradigm of context-dependent sustained inhibition of learned motor response. These data showed a stable and lasting increase in alpha power indicative of enduring active inhibitory processes within the motor cortex (Hummel et al., 2002; Sauseng et al., 2013). Action inhibition involves several brain regions of which, together with the motor cortex, especially prefrontal regions play an important role (Swann et al., 2009; Hwang et al., 2014). Previous studies exploring whole brain activity changes during sustained inhibition of learned motor responses however have revealed strongest changes at the bilateral motor cortices (Hummel et al., 2002) and in the upper alpha band (Swann et al., 2009). Based on this, an engagement of prefrontal regions, as detected by previous studies using Go/Nogo, stroop or non-motor tasks on cognitive control and conflict monitoring (Egner and Hirsch, 2005; Swann et al., 2009), was not expected in this study. In fact, a model of action control by Dillon and Pizzagalli suggests two loops comprising segregated motor areas (cerebellum, thalamus, basal ganglia, prefrontal cortex) which integrate at the level of the primary motor cortex projecting to the spinal cord (Band and van Boxtel, 1999; Dillon and Pizzagalli, 2007). Together this motivated our a priori focusing on M1.

Here we use this motor paradigm of context-dependent executive control of learned complex finger movements in order to investigate age-related differences in alpha oscillations during inhibition.

Groups of young (30 ≤ years) and old (65> years) participants were asked to acquire a complex motor skill. Subsequently, the execution and inhibition of the acquired movement in a context-sensitive manner was required while multi-channel electroencephalogram (EEG) was recorded.

We hypothesized that a cortical correlate of a reduced inhibitiory control with age (as reviewed above), elderly participants will show less alpha power increase during tasks that require motor inhibition compared to young. Additionally we investigated the effect of an overnight consolidation phase on alpha power modulation.

## Materials and methods

### Participants

The study included 30 healthy participants (17 women), of which 15 were aged 25 ± 2.6 years (mean ± SD) and 15 were aged 70 ± 3.2 years (mean ± SD). All volunteers were right-handed as confirmed by the Edinburgh inventory of handedness (Oldfield, 1971). They were naive to the experimental purpose of the study and did not have a history of neurologic disorder. Participants did not play, or had a history of playing, instruments involving rapid finger transitions. All participants were informed about the nature of the experimental procedure and gave their written informed consent for the study. The elderly volunteers were tested for potential memory decline using the Mini Mental State Examination test (Folstein et al., 1975) and were only included if a score above 28 had been achieved [29.5 ± 0.6 (mean ± SD)]. Per group one participant had to be excluded from data analysis due to technical problems during the recording or excessive muscle artifacts in the EEG. The study conforms to “The Code of Ethics” of the World Medical Association (Declaration of Helsinki) and got approved by the Local Ethical Committee of the Medical Association of Hamburg.

### Motor paradigm

We conducted two experimental sessions on two consecutive days. In the first session, participants trained the complex finger-tapping sequences to an overlearned level as described previously (Gerloff et al., 1997; Hummel et al., 2002; Sauseng et al., 2013). One hour later they performed the motor paradigm during EEG-recording. In the second experimental session, 24 h later, the EEG-recording and performance of the motor paradigm were repeated.

#### The motor sequence

**Figure 1:**
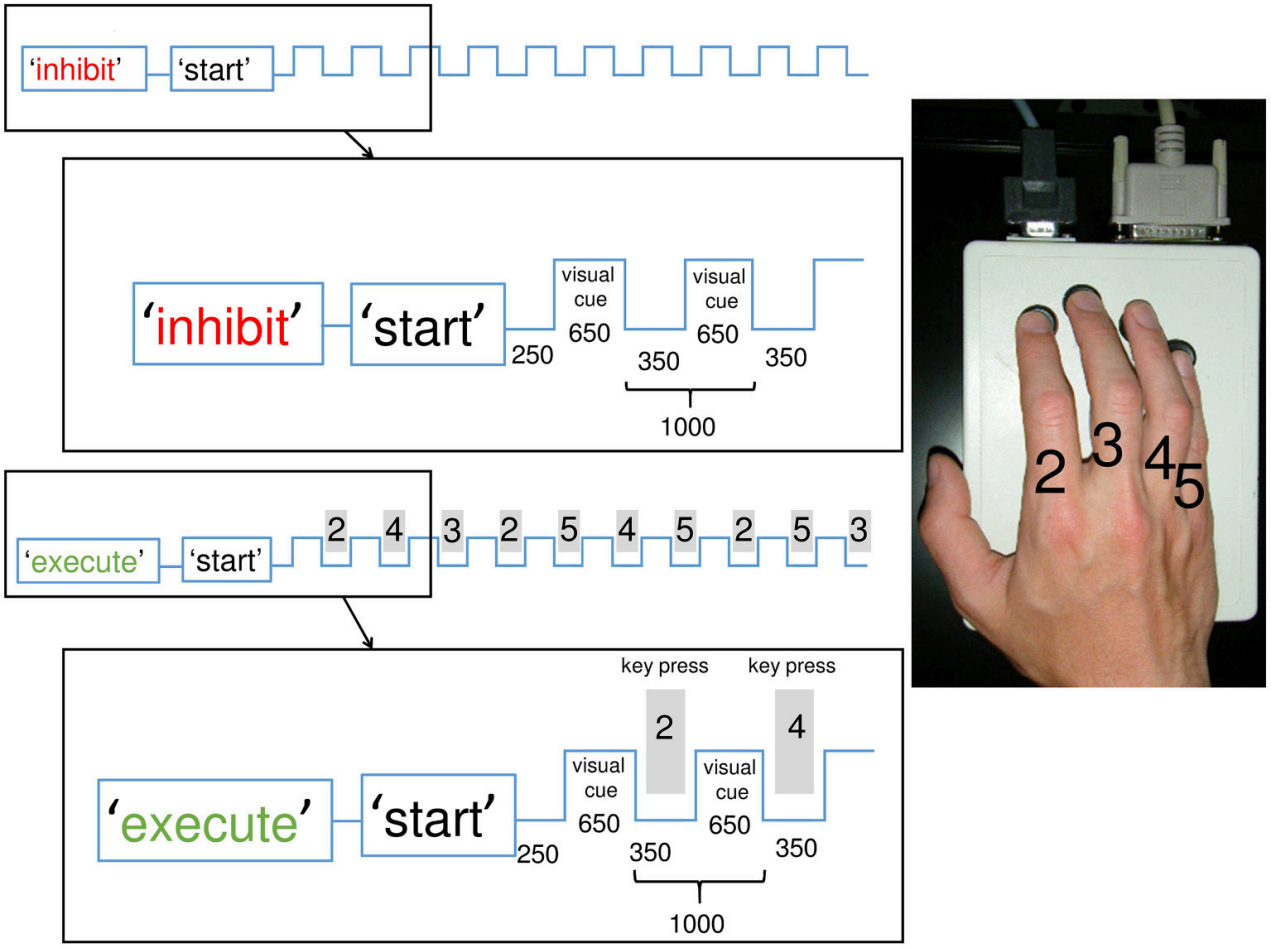
**Illustration of the experimental paradigm with inhibition and execution conditions: Participants had to execute and inhibit the memorized and overlearned sequence each 25 times in a randomized order (50 sequences total) with breaks in between**. The conditions were announced on a screen prior to each block. In the inhibition condition, participants observed the visual cues (symbols) but withheld the reaction of a key press. Each square wave represents a visual cue. In the execution condition, participants responded to the cues (also symbols) with a key-press (gray rectangle) according to the memorized sequence. The sequence consisted of 10 finger taps played at 1 Hz on an electric keyboard. The index was labeled “2,” the middle finger “3,” the ring finger “4,” the little finger “5.” The magnification shows the exact timing (in ms) of the first two steps of each condition. The experiment was conducted 1 h after learning and 24 h later.

The sequence consisted of 10 consecutive finger movements (key presses on an electronic button box) involving four fingers of the right hand in a complex order (2-4-3-2-5-4-5-2-5-3). The numbering of fingers was as follows: index finger = 2, middle finger = 3, ring finger = 4, little finger = 5, see Figure 1. The complex sequence was generated randomly but preventing identical subsequent key presses and matching the occurrence of finger taps in the sequence in order to avoid expectation of frequent finger taps.

#### Learning of the motor sequence

On the first day during a practice session, participants were asked to train the complex motor sequence to a level to be able to perform it 10 times in a row with a metronome pacing at 1 Hz without any errors. Reaching the requested level, the sequence was regarded as “overlearned” according to previous studies (Gerloff et al., 1997; Hummel et al., 2002, 2003). The experiment was on purpose designed to prevent behavioral differences in terms of different error rates between elderly and young participants by both groups performing on ceiling level. Consequently, different spectral characteristics during behavioral inhibition would merely reflect altered cortical processes of sustained inhibition.

### Experimental setup

A similar paradigm has been used in previous studies (Hummel et al., 2002, 2003; Sauseng et al., 2013). Participants sat comfortably in an armchair with the right arm relaxed and positioned on a pillow palm down. Volunteers were asked to perform two experimental conditions: in the “execute” condition, they had to play the memorized sequence according to visual pacing (1 Hz) and in the “inhibit” condition, they had to withhold playing the sequence while attentively watching the same visual pacing. During an experimental session, participants had to execute and inhibit the sequence each 25 times in a randomized order (50 sequences total) with breaks in between. Visual symbols were used as pacing cues (“#,” “&,” “+,” “%”) without any relation to the learned sequence. These symbols were presented on the monitor at a rate of 1 Hz, simply to pace the participants' finger presses. In each trial, prior to the start of a train of visual symbols participants were instructed by a written presentation on the computer screen of whether to execute or withhold the sequential finger movements, see Figure 1. In all conditions, participants looked at a stationary fixation point in the center of the screen to minimize eye movement artifacts. Participants were instructed to perform the sequential finger movements as accurately as possible and to continue without correction movements irrespective of errors they may have made. No feedback was given regarding the correctness of played sequences.

As a baseline, a 5 min pre- and post-experimental EEG was recorded at unconstrained rest with eyes open.

### EEG recording

Continuous EEG was recorded from 63 cephalic active surface electrodes arranged according to the 10-10 system (actiCAP®, Brain Products GmbH, Germany, Gilching). Impedance was kept below 20 kΩ. Using a BrainAmp MR Plus® amplifier (Brain Products GmbH, Germany, Gilching) data were sampled at 1000 Hz, without pre-filtering and referenced to a nose tip electrode. One electrode was mounted below the left eye for EOG-recording. Key presses and visual trigger stimuli were automatically documented with markers in the continuous EEG file. Participants were instructed to avoid eye blinks, swallowing, or any movements other than the required finger movements. EEG was recorded continuously during the experimental session.

### EEG data analysis

Analysis was performed with the FieldTrip package for EEG/MEG data analysis (Oostenveld et al., 2011) on MATLAB Version 7.8.0 (R2009, Mathworks Inc., Massachusetts).

#### Preprocessing

The continuous EEG was high- and low-pass filtered at 2 and 70 Hz, band-stop filtered at 49 to 51 Hz and segmented into epochs of 1000 ms (± 500 ms visual cue onset for the experimental condition); epochs containing artifacts were rejected manually. This was done separately for experimental and rest condition. Trials of the inhibition condition in which participants erroneously pressed a key were also rejected. The pre and post baseline EEG-recordings were pooled to one resting condition. In the young group, 187 ± 61 trials (mean ± SD) and in the elderly group 155 ± 44 trials (mean ± SD) for each condition (execute, inhibit, rest) were left for analysis.

#### Source spectral power analysis

The cross-spectral density matrix at channel level was calculated using fast Fourier transformation and a Hanning taper for a frequency centered at 13 Hz (± 1 Hz) for the inhibition condition and 10 Hz (± 2 Hz) for the execution condition. This frequency was chosen on the basis of the spectral power distribution at scalp level, where in elderly and young participants an increase around 13 Hz in the inhibition condition and a decrease around 10 Hz in the execution condition was found (data not shown). The selected frequency ranges are well in line with previous studies: i.e., in Hummel et al. execution condition showed the most dominant effects from 9 to 13 Hz and the inhibition condition at 11–13 Hz and in Sauseng et al. the inhibition condition showed a strongest effect at 10–13 Hz (Hummel et al., 2002; Sauseng et al., 2013). To extract spectral power at regions of interest, we applied the ELORETA inverse technique (exact low-resolution electromagnetic tomography) in the frequency domain (Pascual-Marqui, 2007). As a forward model we used a standard Boundary Element Method volume conduction model (Oostenveld et al., 2003). The forward model was constructed based on a segmented template MRI brain. Individual electrode positions were determined using the Zebris localization system (CMS20, Zebris, Isny, Germany) and realigned to the template MRI brain. The leadfield for each dipole position on a 4-mm equally spaced grid within the brain volume was computed. This, together with the cross-spectral density matrix, served as a basis to compute a spatial filter. We estimated the power for the different conditions and for every participant individually. To reduce variances of spectral power estimates and to account for inter-participant variability, the derived power estimates were stabilized by logarithmic (log) transformation (Halliday et al., 1995) and expressed as the change of spectral power during inhibition compared to the rest period according to:

$$\text{TR}-\text{Pow}=\text{log}[{\text{Pow}}_{\text{execution/inhibition}}]-\text{log}[{\text{Pow}}_{\text{rest}}]$$

For statistical analyses, MNI coordinates for the regions M1 left and right were a priori defined based on a previous fMRI-experiment using the same paradigm (Hummel et al., 2004). The coordinates of the motor cortices with a 1 cm sphere where chosen (*x* = −29, *y* = −23, *z* = 60, for M1 left (left sensorimotor cortex, LSM), around *x* = 34, *y* = −25, *z* = 63, for M1 right (right sensorimotor cortex, RSM) in MNI coordinates after transformation from Talairach space).

#### Statistical analyses

For statistical analysis of behavioral data (erroneous key presses in the inhibition condition), the non-parametric Wilcoxon rank sum test (Mann-Whitney *U*-test) was used to test differences in error rates across groups and the Wilcoxon signed rank test to test differences in error rates from 1 to 24 h post learning within groups. Differences were considered as significant if *p* < 0.05.

One-sample *T*-tests (for young and elderly participants separately) testing TR-Pow as the dependent variable against zero during the execution and inhibition condition 1 h and 1 day after learning were run to evaluate (i) whether playing the sequence lead to a significant alpha power decrease compared to rest and (ii) whether withholding responses led to significantly higher alpha power compared to rest.

To test our hypothesis that elderly show less inhibitory alpha power during the suppression of a learned motor sequence 24 h after learning, TR-Pow during the inhibition condition 1 day after learning was compared between groups (young vs. elderly) using an independent sample *T*-test. Since TR-Pow is the source spectral power during the execution or inhibition condition normalized by the baseline power, the *T*-tests test the hypothesis that the data come from a distribution with mean zero.

To test our hypothesis of an increase in alpha power specifically in the elderly group over a consolidation phase of 24 h after learning, we performed a repeated measures Analysis of Variance (rmANOVA) with TR-Pow values as dependent variable and with the within-subject factors TIME (1 vs. 24 h) and REGION (M1 left, right) and between subject factors GROUP (young vs. elderly).

Similarly, a rmANOVA with TR-Pow values as dependent variable was performed for the execution condition.

Since TR-Pow is expressed as the change of spectral power during inhibition compared to the rest period, we excluded a difference in baseline upper alpha TR-Pow between the two groups or measurements by performing the same statistical analysis as on the inhibition condition.

All statistical analyses were conducted with SPSS 22.0 (SPSS for Windows 22.0; SPSS, Chicago, IL) or MATLAB Version 7.12.0 (R2011a, Mathworks Inc.).

## Results

### Behavioral data

The frequency of unsuccessful withholding of the finger sequence in the inhibition condition was compared between elderly and young participants as well as 1 and 24 h after learning. Over all participants reached a highly overlearned level of sequence execution. The number of erroneously pressed keys by young participants was at 1 h 0.15 ± 0.28% (mean ± std.) and by elderly participants 0.19 ± 0.19%. At 24 h the young made 0.15 ± 0.19% errors and the elderly 0.08 ± 0.13%. The reduction of errors by the elderly participants from 1 to 24 h was not significant (Wilcoxon signed rank, *W* = 0, *p* = 0.062). There was no significant group difference at neither measurement (Wilcoxon rank sum test, young vs. old 1 h: *U* = 79.5, *p* = 0.401; young vs. old 24 h: *U* = 74, *p* = 0.285). Sequences of the EEG-recording with erroneously pressed keys were omitted.

### Source spectral power analyses

**Figure 2:**
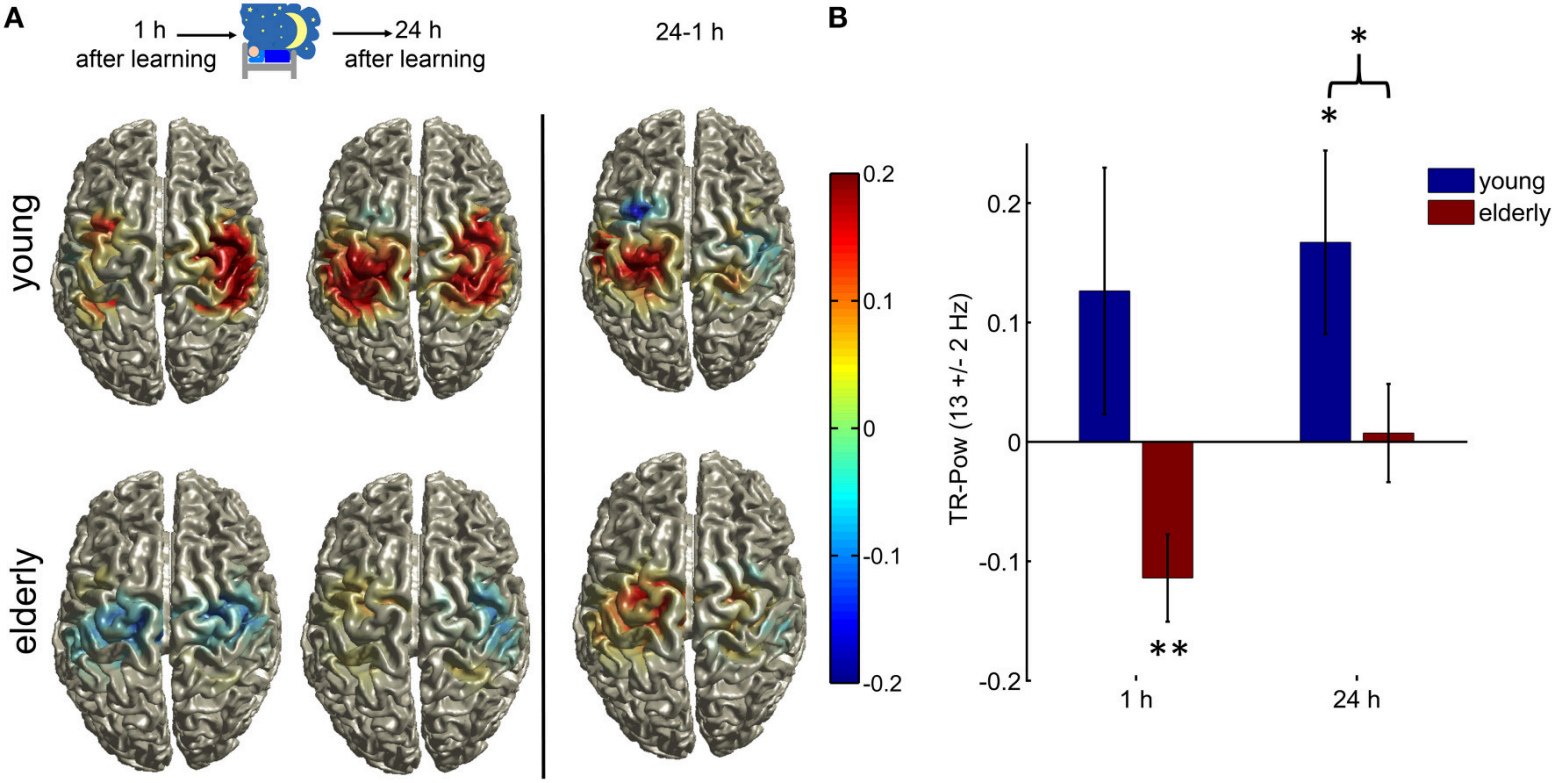
**(A) Topographical illustration of TR-Pow at the sensorimotor cortices in the upper alpha band (12–14 Hz) during the inhibition condition in elderly and young, 1 h after learning and after a one night consolidation phase**. At the right side the difference between the two measurements (24 h—1 h) is plotted. Only TR-Pow around center coordinates of LSM and RSM is shown. **(B)** Bar plots of the relative TR-Pow changes at the left and right motorcortices (averaged) in the upper alpha band (12–14 Hz) during the inhibition condition in both groups at both measurements. Error bars = 1 SEM; (^*^indicates *p* < 0.05, ^**^p < 0.01). The elderly group showed a significant decrease in alpha power similar to the execution condition but to a much smaller extend (one-sample *T*-test old 1 h: *T* = −3.12, *p* = 0.008). The young group showed a significant increase of alpha power at the motor cortices 24 h after learning (one-sample *T*-test young *T* = 2.18, *p* = 0.049). The two groups differed significantly in their generation of alpha rhythm during inhibition 24 h after learning (two-sample *T*-test, *T* = 2.00, *p* = 0.049). See Results section on Source Spectral Power Analyses for the results of a rmANOVA with the within-subject factors TIME and REGION and between subject factors GROUP.

We used the ELORETA technique to estimate source spectral power in the upper alpha band in the group of young and elderly participants separately during the experimental conditions (execution and inhibition) at 1 and 24 h after training. Topographical maps of TR-Pow centered at the sensorimotor cortices are depicted in Figure 2A and Supplementary Figure 1.

In both groups there was a significant alpha power decrease over the bilateral motor cortices during the execution of finger movements at both sessions (one-sample *T*-test young 1 h: *T* = −3.64, *p* = 0.003, 24 h: *T* = −4.58, *p* = 0.005; old 1 h: *T* = −5.67, *p* = 0.0001, 24 h: *T* = −4.96, *p* = 0.0003).

One hour after learning there was an increase in alpha power in the young group, but this did not reach significance (one-sample *T*-test young 1 h: *T* = 1.22, *p* = 0.24). The elderly group showed a significant decrease of alpha power at the motor cortices (one-sample *T*-test old 1 h: *T* = −3.12, *p* = 0.008), similar to the execution condition, but no increase of alpha power as in young. The young group showed a significant increase in alpha power (one-sample *T*-test young 24 h: *T* = 2.18, *p* = 0.049) 24 h after learning whereas in the elderly this was not significant (one-sample *T*-test old 24 h: *T* = 0.18, *p* = 0.86).

**Hypothesis 1:**

The first hypothesis we tested with the present experiment was that elderly show less inhibitory alpha power during the suppression of a learned motor sequence 24 h after learning.

The two groups differed significantly in their generation of alpha rhythm during inhibition 24 h after learning (two-sample *T*-test old vs. young 24 h: *T* = 2.00, *p* = 0.049, Figure 2B). There was no difference in baseline alpha power between the two groups (two-sample *T*-test: *T* = 1.11, *p* = 0.27).

**Hypothesis 2:**

The second hypothesis we tested was, that prior motor memory formation plays an important role for the modulation of inhibitory alpha power, specifically that elderly will not show an increase in upregulation of inhibitory alpha power within a 24 h consolidation phase. Alpha power during the suppression of a learned motor sequence one and 24 h after learning was analyzed in elderly and young.

We performed a rmANOVA with TR-Pow values as the dependent variable and with the within-subject factors TIME and REGION and between subject factors GROUP and found significant main effects for the factor TIME [*F*_(1, 26)_ = 4.80, *p* = 0.038] and the factor GROUP [*F*_(1, 26)_ = 4.63, *p* = 0.041]. There was a larger alpha power increase after 24 h and in young participants, the alpha power increase was stronger. No other main effect or interaction reached significance [factor REGION *F*_(1, 26)_ = 0.18, *p* = 0.68, interaction TIME × REGION *F*_(1, 26)_ = 1.81, *p* = 0.19, interaction TIME × GROUP *F*_(1, 26)_ = 1.19, *p* = 0.29, interaction REGION × GROUP *F*_(1, 26)_ = 0.35, *p* = 0.56, interaction TIME × REGION × GROUP *F*_(1, 26)_ = 1.10, *p* = 0.30]. There was no difference in baseline alpha power between groups across time {rmANOVA factor GROUP [*F*_(1, 26)_ = 0.91, *p* = 0.35], TIME [*F*_(1, 26)_ = 3.82, *p* = 0.06], factor REGION [*F*_(1, 26)_ = 0.28, *p* = 0.60], TIME × REGION [*F*_(1, 26)_ = 0.17, *p* = 0.68], TIME × GROUP [*F*_(1, 26)_ = 0.19, *p* = 0.67], REGION × GROUP [*F*_(1, 26)_ = 0.24, *p* = 0.63], TIME × REGION × GROUP [*F*_(1, 26)_ = 0.96, *p* = 0.34]}.

This time-dependent increase in spectral power was specific to the upper alpha band. A control analysis on scalp level data in the beta band (23–27 Hz) showed no significant increase over time [main factor TIME *F*_(1, 26)_ = 0.76, *p* = 0.39] and no TIME × GROUP interaction [*F*_(1, 26)_ = 3.11, *p* = 0.09] whereas a significant group difference was evident [factor GROUP *F*_(1, 26)_ = 6.65, *p* = 0.016; see Supplementary Figure 2, for details].

To check if there is also a time-dependent change of the alpha power decrease we calculated the same rmANOVA design for the execution condition. The rmANOVA with the within-subject factors TIME (1 vs. 24 h) and REGION (M1 left, right) and between-subject factors GROUP (young vs. elderly) showed no significant effects [factor TIME *F*_(1, 26)_ = 0.08, *p* = 0.78, factor GROUP *F*_(1, 26)_ = 0.80, *p* = 0.38, factor REGION *F*_(1, 26)_ = 0.71, *p* = 0.41, interaction TIME × REGION *F*_(1, 26)_ = 0.35, *p* = 0.56, interaction TIME × GROUP *F*_(1, 26)_ = 2.54, *p* = 0.12, interaction REGION × GROUP *F*_(1, 26)_ = 0.65, *p* = 0.43, interaction TIME × REGION × GROUP *F*_(1, 26)_ = 0.30, *p* = 0.59].

## Discussion

In previous studies, it has been shown that during withholding of a learned motor response, the EEG alpha amplitude over sensorimotor regions increases. Based on known deficits in executive functioning in the elderly (Bedard et al., 2002; Gazzaley and D'Esposito, 2007; Anguera and Gazzaley, 2012), we hypothesized, that elderly individuals will not show an increase of alpha power as a cortical correlate of behaviorally successful inhibition.

During withholding of the motor response the group of elderly participants did not show an increase in task-related alpha power as a correlate of inhibitory control. This was in contrast to young volunteers: at both evaluation points, 1 h and 1 day after training, there was a clear difference of alpha power between elderly and young subjects. One hour after learning, in elderly participants we even found signs for motor preparation instead of motor inhibition, i.e., alpha power decrease at the sensorimotor cortices. Similarly, in a study by Vallesi et al. (2010), the authors showed correlates of response preparation in a no-go situation in old compared to young individuals. In regard to previous findings in comparable tasks, e.g., by Hummel et al. (2002), the present results point to a significant deficit in generating neurophysiological correlates of context-dependent motor-response inhibition in elderly individuals.

Increased alpha power has initially been described as a correlate of idling of cortical areas (Pfurtscheller et al., 1996). However, higher spontaneous alpha power is associated with (i) a higher threshold in stimulus detection (Romei et al., 2008; Busch et al., 2009), (ii) smaller motor evoked potential (MEP) elicited by transcranial magnet stimulation (TMS) on the primary motor cortex (Sauseng et al., 2009), and (iii) decreased performance on perceptual and motor tasks (Hanslmayr et al., 2007; Mazaheri et al., 2009); suggesting alpha oscillations as a correlate of functional inhibition. Recent observations further indicate that alpha power is increased during tasks requesting inhibitory control of behavior. For example, in situations where subjects have to withhold or control a response, or suppress irrelevant information, increases in alpha power have been observed; likewise over visual areas contralateral to unattended locations (Jensen et al., 2002; Schack and Klimesch, 2002; Busch and Herrmann, 2003; Cooper et al., 2003; Klimesch et al., 2007; Jensen and Mazaheri, 2010; Thut et al., 2011). In tasks requiring a sustained level of inhibition in the motor domain a stable increase in alpha power over the sensorimotor cortices was found during withholding of a learned, automated motor program (Hummel et al., 2002, 2004; Sauseng et al., 2013). This inhibitory state leads to a significant reduction in MEP amplitudes tested with TMS, suggesting a reduced corticospinal excitability of the sensorimotor cortex, below resting state (Hummel et al., 2002). Based on this work, a framework of a process-specific alpha theory of inhibition was suggested by Klimesch et al. assuming that alpha activity is a neural signature of inhibitory top-down control (Klimesch et al., 2007). Thus, the present lack of increase of alpha power in the elderly participants points toward a deficit in inhibitory control mechanisms in elderly humans.

From a behavioral point of view, impaired ability to inhibit inappropriate responses is prepotent and has been demonstrated in old adults in different studies (Hasher et al., 1991, 1999; Gottlob et al., 2007; Andrés et al., 2008). As our main interest was to evaluate underlying cortical mechanisms of inhibitory control in the elderly, the paradigm was designed to avoid confounding behavioral differences between groups by achieving comparable error rates across groups. Furthermore, during the inhibition condition, no response was required and the subjects received the information not to move beforehand (several seconds before the start of a sequence). Thus, the present task does not require a fast stop of movement preparation in a Go/No-Go fashion, consequently there were very low false alarm rates (< 1%). The present finding of impairment of cortical correlates of inhibition is likely to be one relevant reason for age-related deficits in cognitive control. Whether the demonstrated impairment has behavioral consequences in daily life has to be addressed in upcoming studies.

Models on cognitive action control focus on two main loops: one comprising cortical areas such as the pre cortex, the basal ganglia and the thalamus where responses are selected and inhibited; and another loop comprising the cerebellum, thalamus, basal ganglia, where activity of the first loop is fine-tuned (Band and van Boxtel, 1999; Dillon and Pizzagalli, 2007). Both loops integrate at the level of the primary motor cortex projecting to the spinal cord. According to these models, the same cortical and subcortical structures are responsible for the initiation and the inhibition of an action. Thus, the driving mechanism for the alpha increase as a correlate of inhibitory control must be within the structures that encode the inhibited motor memory trace formed during training, suggested by recent work (Hummel et al., 2002, 2004; Sauseng et al., 2013).

Among the brain regions most severely affected by aging, the prefrontal cortex is particularly vulnerable (Burke and Barnes, 2006) to significant loss of gray and white matter volume in humans (de Brabander et al., 1998; Decarli et al., 2005; Fotenos et al., 2005; Raz et al., 2005). The important question whether the observed increase in alpha power during the withholding of a response is mediated via corticocortical projections originating in the prefrontal cortex is open. In a first approach, Sauseng et al. identified a frontocentral network of decreased phase coherence during the described inhibition task in the alpha frequency range in young adults (Sauseng et al., 2013). This finding supports the relevance of the prefrontal cortex, and its interactions for inhibitory cognitive control processes (Aron, 2007).

However, not only for the prefrontal cortex but also for the sensorimotor cortex, age-related changes have been reported. Besides structural changes, also age-related alterations of intracortical neurotransmission in the motor cortex, i.e., intracortical inhibition and facilitation, have been described during the resting-state (Peinemann et al., 2001; Kossev et al., 2002; Smith et al., 2009; McGinley et al., 2010). The reduced resting-state inhibitory tone in the elderly was associated with reduced capacities of modulating inhibitory (i.e., GABA-ergic) activity related to motor actions (Heise et al., 2013). These changes were correlated with age and performance in fine skilled and fast movements, pointing to differences in inhibitory circuits at the sensorimotor cortices in elderly compared to young adults. At the microstructural level, age-related changes in animals have shown a reduction of inhibitory synapses in the sensorimotor cortex, further supporting the idea of an age-dependent deficit in the intrinsic inhibitory circuitry (Poe et al., 2001).

Taking these findings together, the present results of reduced inhibitory alpha activity in elderly individuals fit well in the concept of age-related impairment of inhibitory neurotransmission. This might be based on different mechanisms such as deficits in the generation and modulation of local inhibitory mechanisms in the sensorimotor cortices, reduced recruitment of prefrontal subregions involved in top-down executive control and impaired cortico-cortical interactions within the motor control network in the aged brain.

We predefined the frequency ranges for the execution and inhibition condition according to strongest deflections in spectral power distribution at scalp level common in elderly and young patients. The detected frequency ranges are well in line with previous studies (Hummel et al., 2002; Sauseng et al., 2013). Predefining frequency ranges according to individual or group peaks is commonly done. This increases the effect size while also potentially introducing a bias toward false positives. The latter especially if the selected frequency ranges differ between samples tested against each other. However, our hypotheses are focused on group and time differences and not condition differences, thus a false positive results due to preselection is avoided.

Within the present experimental design, we also addressed the relevance of time for the development of cortical inhibitory control of trained and memorized motor responses by repeating the task at 1 and 24 h after learning. In young participants, a significant increase in alpha power was seen 24 h after learning, whereas 1 h after learning the alpha power increase was apparent but did not yet significantly differ from baseline. Elderly participants, in contrast, did not show a significant increase in alpha power at the primary motor cortices at neither measurement. However, at 24 h the decrease of alpha power during the inhibition condition was much less than after only 1 h. In previous studies using a similar paradigm (Hummel et al., 2002; Sauseng et al., 2013) the learning of the motor sequence and the experimental testing where separated by 24 h in order to test retrieval and inhibition of the motor act from long-term memory. It was suggested, that the formation of an “external cue-motor output” during the training session and subsequent overnight consolidation is an essential prerequisite for inhibitory control. The present study addressed this and the results suggest an importance of motor memory formation for the observed inhibitory alpha power increase. This finding emphasizes the involvement of motor plastic changes at the primary motor cortex within the 24 h interval after extensive learning and retrieval of the motor sequence (Wiestler and Diedrichsen, 2013). In rats Xu et al. detected rapid (starting within an hour, peaking 24 h after learning) formation of postsynaptic dendritic spines on the output pyramidal neurons in the contralateral motor cortex after motor-skill learning (Xu et al., 2009). It is likely that within the 24 h interval plastic changes involving synaptic strengthening take place which facilitate the execution of the motor sequence and that these training-induced motor memory traces need relatively stronger suppressive signal during the inhibition condition. Whether the elderly participants just need more time to develop the inhibitory control represented by alpha power increase is an open question that has to be addressed in upcoming studies with more measurement time points after learning. Looking at the groups separately, we found that TR-Pow at the upper alpha band during inhibition in young individuals essentially stays the same over time [one-sample *T*-test (24 h—1 h): *T* = −0.86, *p* = 0.39] whereas in elderly, the increase in TR-Pow over night was significant (*T* = −4.16, *p* = 0.0002). A reduced synapse number in aged brains could be a reason for plastic changes occurring slower in elderly since a sufficient amount of cooperatively active synapses initiating a network modification is less likely reached (Burke and Barnes, 2006).

In conclusion, the up-regulation of alpha power over the motor cortices, as an indicator of an active inhibitory control process of motor behavior, is differentially expressed in young and elderly participants. Elderly people show a reduced capacity of generating this inhibitory state, probably due to altered network properties between frontal executive and the sensorimotor cortices. Consolidation of a learned motor sequence leads to enhanced inhibitory alpha rhythms in young and to a lower extent in elderly, possibly due to the overnight strengthening, thus consolidation of the acquired motor memory trace.

## Conflict of interest statement

The authors declare that the research was conducted in the absence of any commercial or financial relationships that could be construed as a potential conflict of interest.

## Supplementary material

Supplementary Figure 1 **(A)** Topographical illustration of TR-Pow at the left and right motorcortices in the alpha band (8–12 Hz) during the execution condition in elderly (right) and young (left). Only TR-Pow around center coordinates of LSM and RSM is shown. The two measurements are pooled due to no significant change over time [*F*_(1, 26)_ = 0.079, *p* = 0.78]. **(B)** Bar plots of the relative TR-Pow changes at the left and right motorcortices (averaged) in the alpha band (8–12 Hz) during the execution condition in both groups at both measurements. Error bars = 1 SEM; one-sample *T*-test; ^**^indicates *p* < 0.01, ^***^*p* < 0.001).

Supplementary Figure 2 **Bar plots of the relative TR-Pow changes during the inhibition condition at scalp level**. TR-Pow at the left and right motor cortices (electrodes C3, C4, CP3, CP4, FC3, FC4) are averaged in the beta band (23–27 Hz) in both groups at both measurements. Error bars = 1 SEM; (^**^indicates *p* < 0.01, ^***^*p* < 0.001). In the young group at 24 h after learning, the beta TR-Pow was significantly increased (one-sample *T*-tests: young 24 h: *T* = 4.74, *p* = 0.0004). The two groups differed significantly in their generation of beta rhythm 24 h after learning during inhibition (two-sample *T*-test, *T* = 2.98, *p* = 0.006). See Results section on Source Spectral Power Analyses for the results of a rmANOVA with the within-subject factors TIME and REGION and between subject factors GROUP.

## Abbreviations

EEG: electroencephalogram
ELORETA: exact low-resolution electromagnetic tomography
MNI: Montreal Neurological Institute
rmANOVA: repeated measures Analysis of Variance
TR-Pow: Task-related spectral power
LSM: left sensorimotor cortex
RSM: right sensorimotor cortex
MEP: motor evoked potential
SEM: standard error of the mean
TMS: transcranial magnetic stimulation.
