# Supplementary figures and images for: Alpha oscillatory correlates of motor inhibition in the aged brain
Source: Front Aging Neurosci. 2015 Oct 13;7:193. doi: 10.3389/fnagi.2015.00193 (PMC4602091; doi:10.3389/fnagi.2015.00193)

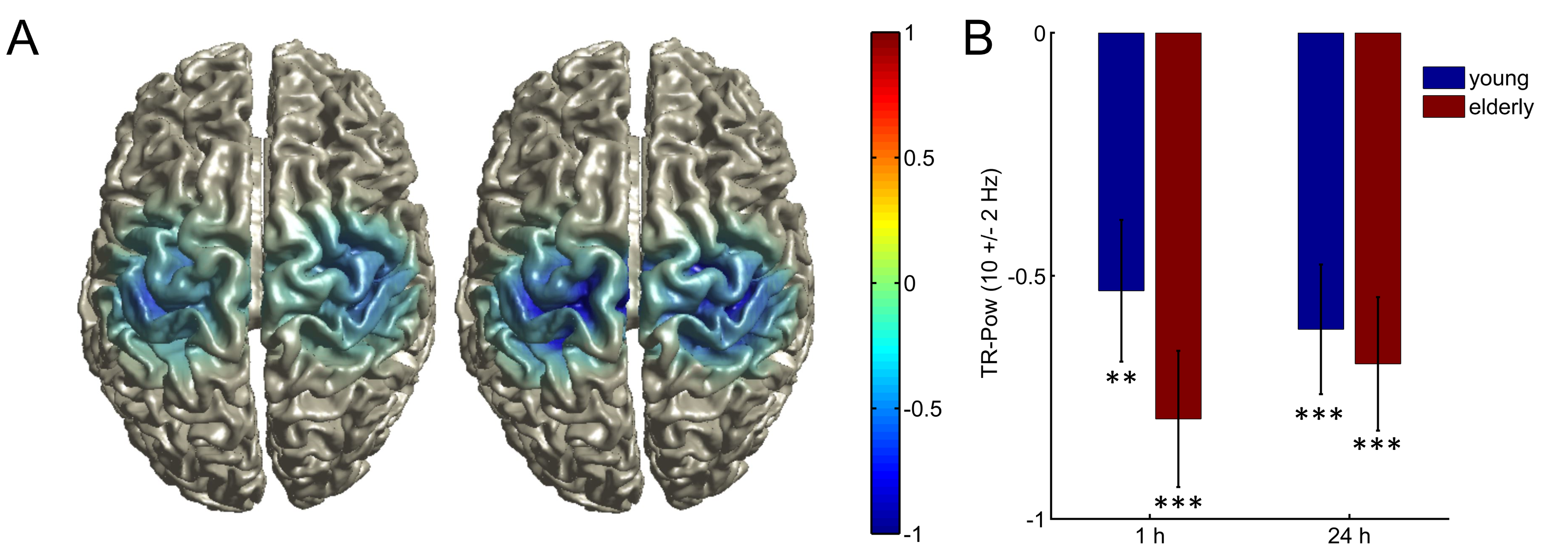

Supplement: Supplementary Figure 1 — (A) Topographical illustration of TR-Pow at the left and right motorcortices in the alpha band (8–12 Hz) during the execution condition in elderly (right) and young (left). Only TR-Pow around center coordinates of LSM and RSM is shown. The two measurements are pooled due to no significant change over time [F(1, 26) = 0.079, p = 0.78]. (B) Bar plots of the relative TR-Pow changes at the left and right motorcortices (averaged) in the alpha band (8–12 Hz) during the execution condition in both groups at both measurements. Error bars = 1 SEM; one-sample T-test; **indicates p < 0.01, ***p < 0.001). [file Image1.TIF]

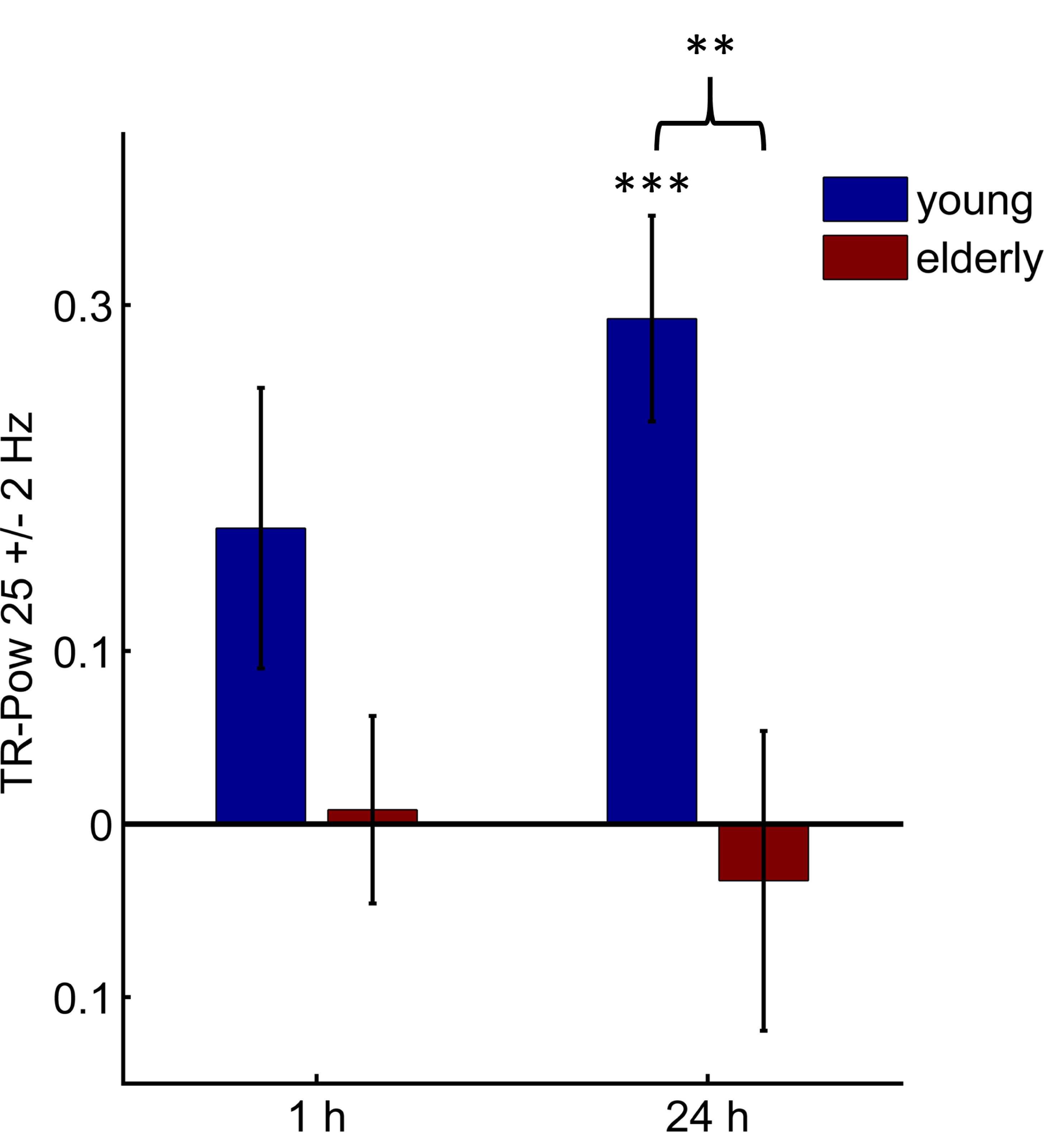

Supplement: Supplementary Figure 2 — Bar plots of the relative TR-Pow changes during the inhibition condition at scalp level. TR-Pow at the left and right motor cortices (electrodes C3, C4, CP3, CP4, FC3, FC4) are averaged in the beta band (23–27 Hz) in both groups at both measurements. Error bars = 1 SEM; (**indicates p < 0.01, ***p < 0.001). In the young group at 24 h after learning, the beta TR-Pow was significantly increased (one-sample T-tests: young 24 h: T = 4.74, p = 0.0004). The two groups differed significantly in their generation of beta rhythm 24 h after learning during inhibition (two-sample T-test, T = 2.98, p = 0.006). See Results section on Source Spectral Power Analyses for the results of a rmANOVA with the within-subject factors TIME and REGION and between subject factors GROUP. [file Image2.TIF]
